# 81030 Utilizing a synergistic drug combination to target relapsed/refractory FLT3 mutant AML

**DOI:** 10.1017/cts.2021.658

**Published:** 2021-03-30

**Authors:** LaQuita Jones, Katelyn Melgar, Scott Hoyt, Mark Wunderlich, Eric O’Brien, John Perentesis, Craig Thomas, Daniel Starczynowski

**Affiliations:** 1 Cincinnati Children’s Hospital Medical Center; 2 National Center for Advancing Translational Sciences

## Abstract

ABSTRACT IMPACT: This work will help to understand a novel therapeutic approach to a common type of acute myeloid leukemia. OBJECTIVES/GOALS: FMS-like tyrosine kinase 3 (or FLT3) mutations occur in ˜30% of acute myeloid leukemia (AML) cases. FLT3 tyrosine kinase domain (TKD) mutations are particularly important in relapsed/refractory FLT3 mutant AML, which portends poor prognosis. This study describes a therapeutic approach to overcoming resistance conferred by FLT3-TKD mutations. METHODS/STUDY POPULATION: To understand the efficacy of a novel type 1 FLT3 inhibitor (NCGC1481), as a monotherapy and combination therapy, several assays were utilized to interrogate functionality of these therapies. Cell lines and patient samples containing aspartate 835 to tyrosine mutations (D835Y, the most common TKD alteration) and phenylalanine 691 to leucine (F691L) were utilized to examine the effects of NCGC1481 with and without other targeted therapies like MEK inhibitors. Specifically, assays measuring viability, cell death using flow cytometry, in vitro clonogenicity, cellular signaling, and xenograft survival were examined in these FLT3-TKD AML models. Synergy was also measured using well described methods, which also allowed for appropriate dose finding for drug combination experiments. RESULTS/ANTICIPATED RESULTS: Our novel type 1 FLT3 inhibitor (NCGC1481) was particularly effective in the most common FLT3 TKD mutant, D835Y. NCGC1481 reduced viability and cell signaling, while also inducing cell death and prolonging xenograft survival in the FLT3-D835Y model system. In contrast, clinically approved FLT3 inhibitors were less effective at suppressing AML cells expressing FLT3-D835Y. In the case of FLT3-F691L, most of the FLT3 inhibitors tested, including NCGC1481, suppressed canonical FLT3 signaling, but did not significantly reduce viability or leukemic clonogenicity. However, when NCGC1481 was combined with other targeted agents like MEK inhibitors, at synergistic doses, eradication of the FLT3-F691L AML clone was substantially increased. DISCUSSION/SIGNIFICANCE OF FINDINGS: In AML, response to FLT3 inhibitor therapy is often short-lived, with resistance sometimes occurring via FLT3-TKD mutations. Given the dismal prognosis of relapsed FLT3 mutant AML, novel therapies are necessary. This study describes efficacy of a novel FLT3 inhibitor, along with its synergistic activity when combined with other targeted agents.

